# Modified Ca_v_1.4 Expression in the *Cacna1f^nob2^* Mouse Due to Alternative Splicing of an ETn Inserted in Exon 2

**DOI:** 10.1371/journal.pone.0002538

**Published:** 2008-07-02

**Authors:** Clinton J. Doering, Renata Rehak, Stephan Bonfield, Jean B. Peloquin, William K. Stell, Silvina C. Mema, Yves Sauvé, John E. McRory

**Affiliations:** 1 Hotchkiss Brain Institute, University of Calgary, Calgary, Canada; 2 Cell Biology and Anatomy / Surgery, University of Calgary, Calgary, Canada; 3 Lions Centre for Retinal Degeneration Research, University of Calgary, Calgary, Canada; 4 Department of Ophthalmology, University of Alberta, Edmonton, Canada; Vrije Universiteit Amsterdam, Netherlands

## Abstract

The *Cacna1f^nob2^* mouse is reported to be a naturally occurring null mutation for the Ca_v_1.4 calcium channel gene and the phenotype of this mouse is not identical to that of the targeted gene knockout model. We found two mRNA species in the *Cacna1f^nob2^* mouse: approximately 90% of the mRNA represents a transcript with an in-frame stop codon within exon 2 of CACNA1F, while approximately 10% of the mRNA represents a transcript in which alternative splicing within the ETn element has removed the stop codon. This latter mRNA codes for full length Ca_v_1.4 protein, detectable by Western blot analysis that is predicted to differ from wild type Ca_v_1.4 protein in a region of approximately 22 amino acids in the N-terminal portion of the protein. Electrophysiological analysis with either mouse Ca_v_1.4^wt^ or Ca_v_1.4^nob2^ cDNA revealed that the alternatively spliced protein does not differ from wild type with respect to activation and inactivation characteristics; however, while the wild type N-terminus interacted with filamin proteins in a biochemical pull-down experiment, the alternatively spliced N-terminus did not. The *Cacna1f^nob2^* mouse electroretinogram displayed reduced b-wave and oscillatory potential amplitudes, and the retina was morphologically disorganized, with substantial reduction in thickness of the outer plexiform layer and sprouting of bipolar cell dendrites ectopically into the outer nuclear layer. Nevertheless, the spatial contrast sensitivity (optokinetic response) of *Cacna1f^nob2^* mice was generally similar to that of wild type mice. These results suggest the *Cacna1f^nob2^* mouse is not a *CACNA1F* knockout model. Rather, alternative splicing within the ETn element can lead to full-length Ca_v_1.4 protein, albeit at reduced levels, and the functional Ca_v_1.4 mutant may be incapable of interacting with cytoskeletal filamin proteins. These changes, do not alter the ability of the *Cacna1f^nob2^* mouse to detect and follow moving sine-wave gratings compared to their wild type counterparts.

## Introduction

Influx of calcium through voltage-gated calcium channels (VGCCs) leads to excitation-contraction coupling, excitation-transcription coupling, neurotransmitter release, and programmed cell death. Disorders of synaptic transmission are thought to be instrumental in two forms of human X-linked congenital stationary night blindness (CSNB): the “incomplete” form (iCSNB, or CSNB2), in which rod- and cone-driven electroretinogram (ERG) responses are reduced in amplitude, but oscillatory potentials (OPs) can be recorded; and the “complete” form (cCSNB, or CSNB1), in which rod-driven ERG responses are greatly reduced or absent but cone-driven responses are relatively well preserved, and OPs are rarely recorded. Mutations within the *CACNA1F* gene coding for Ca_v_1.4 L-type calcium channels have been identified as one cause of CSNB2 [1], [2], as well as X-linked cone-rod dystrophy (CORDX3) [3] and Åland Island eye disease [4]. Over seventy CSNB2 nonsense and missense mutations have been identified (for example, [5]–[12], several of which have been shown to alter the biophysical properties of the channels [13]–[19]; reviewed in [20]).

Knockout of CACNA1F protein in mice following insertion of a self-excising Cre-lox-neo cassette into exon 7 results in an in-frame premature stop codon (G305X) in the Ca_v_1.4 protein [21]. These *Cacna1f^G305X^* mice are characterized by complete loss of the b-wave and oscillatory potentials of the electroretinogram (ERG), absence of cone-driven visually-evoked activity in the superior colliculus, >90% reduction in calcium influx in photoreceptors, and disrupted retinal morphology with loss of photoreceptor synapses and sprouting of horizontal and bipolar cell dendrites into the outer nuclear layer [21]. *Cacna1f^G305X^* mice, therefore, resemble CSNB1 patients in their lack of cone-driven functions. More recently, a second mouse model said to be null for *CACNA1F* has been described [22]. This *Cacna1f^nob2^* mouse arose by spontaneous insertion of a transposable element (ETn) into exon 2, which is predicted to produce an in-frame premature stop codon. Interestingly, while the *Cacna1f^nob2^* mouse also displays disrupted retina morphology similar to that of the *Cacna1f^G305X^* mouse, the ERG of the *Cacna1f^G305X^* mouse is more similar to that of CSNB2 patients, being characterized by a reduced b-wave and oscillatory potentials while cone-driven responses are maintained.

The ETn element responsible for the *Cacna1f^nob2^* mouse belongs to a family of early retrotransposons, approximately 5600 base pairs in length, which are transcribed during embryogenesis [23]–[25]. While they often are a source of mutations by insertion into the coding frame of genes, their characteristic long terminal repeat regions allow them to be alternatively spliced. This property can result in transcription of the gene into which they were inserted, at reduced levels [26], [27]. We hypothesized that the difference in phenotypes of the two mouse models was the result of alternative splicing of the ETn element, which would allow some transcription and synthesis of Ca_v_1.4 protein. We have found two mRNA species in the *Cacna1f^nob2^* mouse, one of which encodes an in-frame stop codon, and another in which the stop codon is missing as a result of splicing within the ETn; as a result, full-length protein was detectable by Western blotting using an antibody raised against the C-terminus of the α_1F_ channel subunit (Ca_v_1.4) protein. The alternatively spliced protein did not differ from the wild type protein with respect to activation and inactivation characteristics in an expression system; however, unlike the wild type protein N-terminus, the alternatively spliced N-terminus did not bind to cytoskeletal filamin proteins. Interestingly, while the outer retinal layers of the *Cacna1f^nob2^* mouse were significantly disorganized, the optokinetic response was not dramatically different from that of wild type mice.

## Results

### Two mRNA species are detected in the Cacna1f^nob2^ mouse

**Figure 1:**
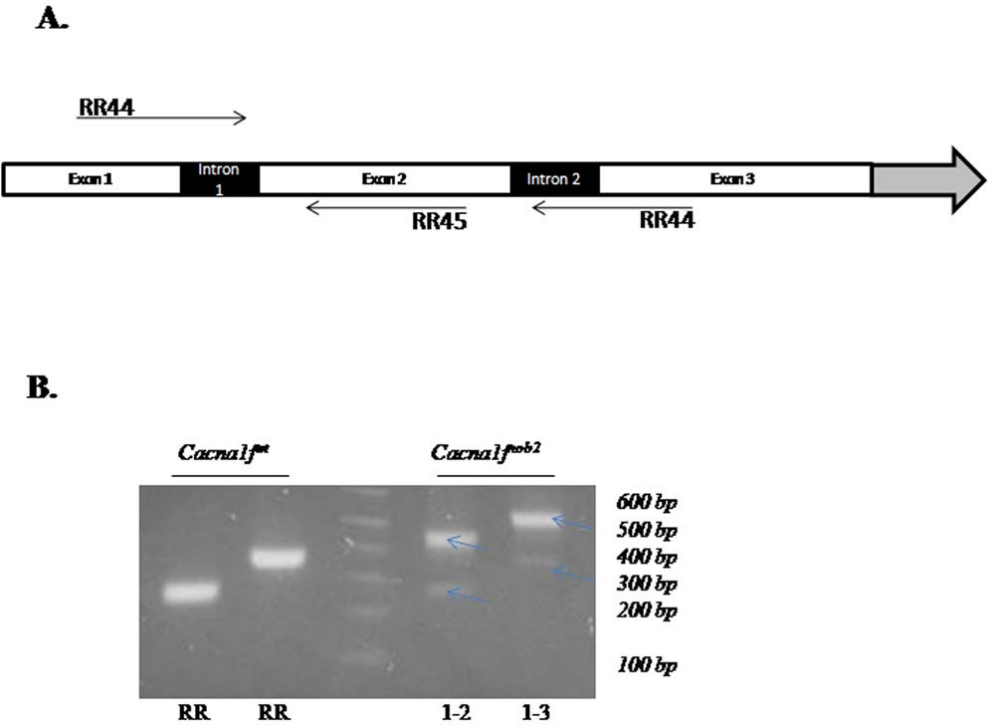
RT-PCR analysis of *Cacna1f^wt^* and *Cacna1f^nob2^* mice. A. Schematic representation of the location of PCR primers used. Primers RR44, 45, and 46 were used for RT-PCR reactions; primers RR50, 51, 52, and 53 were used for genomic PCR reactions. B. Agarose gel depicting RT-PCR reaction products for mRNA isolated from *Cacna1f^wt^* and *Cacna1f^nob2^* mice. Regardless of the primer pair used, only a single band is detected using mRNA from *Cacna1f^wt^* mice. Using mRNA from *Cacna1f^nob2^* mice, however, two bands are visible (see arrows). The relative intensities of the fluorescence signals indicate that the larger-M_r_ band accounts for ∼90%, and the smaller-M_r_ band for ∼10%, of the total mRNA.

**Figure 2:**
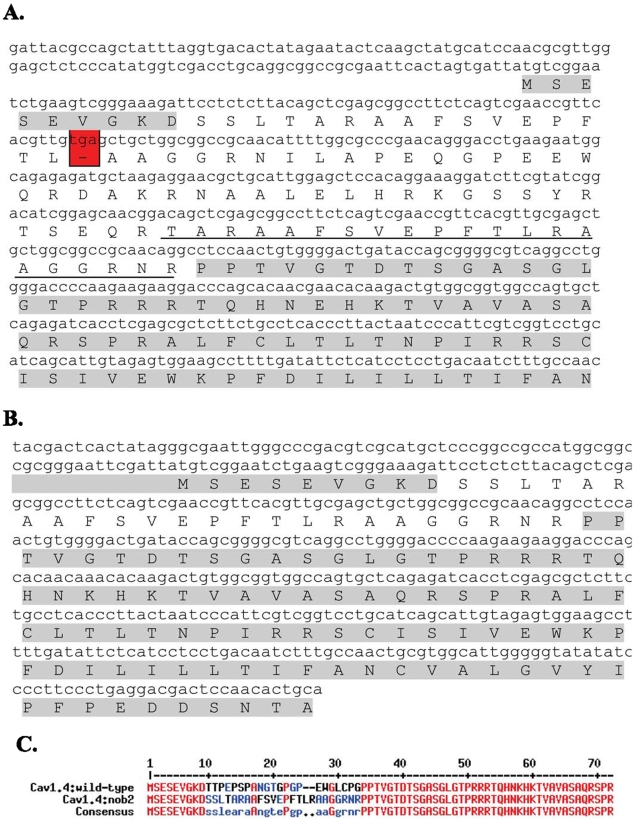
Sequencing results of *Cacna1f^wt^* and *Cacna1f^nob2^* mice. A. Sequencing results from the larger-M_r_, more intense band. Sequences are given for the DNA (small letters) and corresponding protein (capital letters); the sequence corresponding to wild type Ca_v_1.4 protein is highlighted in grey. The ETn transposable element is inserted after the ninth Ca_v_1.4 amino acid, and encodes an in-frame stop codon (highlighted in red) which is predicted to result in truncation of the Ca_v_1.4 protein after only 25 amino acids. The underlined ETn sequence highlights a repetitive sequence (compare to the beginning of the ETn sequence, beginning at amino acid number 4). B. Same as A., but for the smaller-M_r_, less intense band. Note that the inserted ETn element is shorter, and the in-frame stop codon is missing. C. Alignment of the predicted N-terminal amino acid sequences of wild type Ca_v_1.4 protein and Ca_v_1.4 protein encoded in the smaller-M_r_ band. A region of approximately 22 amino acids differs in the N-termini of the two clones.

Total RNA from the eyes of *Cacna1f^wt^* and *Cacna1f^nob2^* mice was analyzed by RT-PCR. As shown in Figure 1.B, while only a single band was detected in *Cacna1f^wt^* mice with either primer combination (RR44+RR 45, or RR44+RR46), two bands were detected in *Cacna1f^nob2^* mice (see table 1 for primers). Using the fluorescence intensity of the bands to assess relative quantity, we estimated that ∼90% of the mRNA is accounted for by the larger (M_r_) band, and ∼10% by the smaller (M_r_) band (consistent results from three separate reactions/gels). The cDNA bands were subsequently isolated and sequenced; the larger and more intense band corresponded to the CACNA1F-encoding sequence containing the ETn transposable element with an in-frame stop codon (Figure 2A), whereas the smaller and less intense band corresponded to the CACNA1F-encoding sequence containing the shorter ETn element that lacks the in-frame stop codon (Figure 2B). Genotyping of these mice, along with genomic DNA samples purchased from Jackson Laboratory, confirmed that our *Cacna1f^nob2^* mice were identical in genotype to the original *Cacna1f^nob2^* mouse line, as described previously [22]. An alignment of the predicted protein sequences corresponding to wild type Ca_v_1.4 (hereafter referred to as Ca_v_1.4^wt^) and Ca_v_1.4 from the smaller size band (hereafter referred to as Ca_v_1.4^nob2^) is provided in Figure 2C.

**Table 1:** Primer sequences.

Primer Name	Primer Sequence (5′→3′)	Primer Use
RR44	atgtcggaatctgaagtcgg	RT-PCR (Figure 1A)
RR45	caatgctgatgcaggaccg	
RR46	gcagtgttggagtcgtcctc	
CDRR69	ggatccaaatgtcggaatctgaagtcg	N-terminus amplification
CDRR70	gaattctcacttccactctacaatgctcatgc	

**Figure 3:**
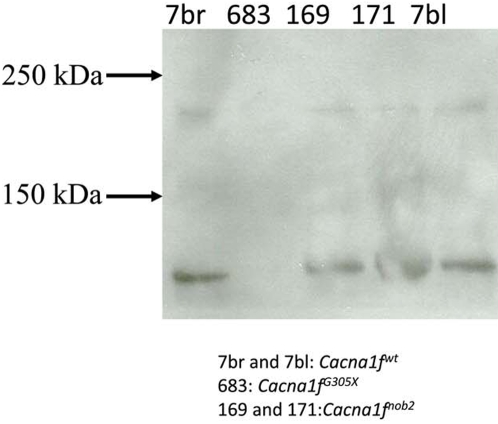
Western blot of spleen samples from *Cacna1f^wt^*, *Cacna1f^nob2^*, and *Cacna1f^G305X^* mice probed with a Ca_v_1.4-specific antibody directed against the C-terminus of the channel. Full-length protein is visible in all lanes except for *Cacna1f^G305X^*.

To test for Ca_v_1.4 protein in the *Cacna1f^nob2^* mouse, we probed lysates from spleen with a Ca_v_1.4-specific antibody [16]. As shown in Figure 3, a band near 230 kDa was detected in samples from both *Cacna1f^wt^* and *Cacna1f^nob2^* mice, but not from *Cacna1f^G305X^* mouse. A lower-M_r_ band (<150 kDa) was also detectable in extracts from all mouse strains tested, except *Cacna1f^G305X^*; this band likely is a truncated form of the wild type protein, as is characteristic of other L-type calcium channel proteins [28].

### Cacna1f^nob2^ mouse has a selective b-wave defect

These results indicated that a full-length Ca_v_1.4 channel (corresponding to the mRNA sequence from the smaller M_r_, less intense band in Figure 1B) could be present in the *Cacna1f^nob2^* mouse. This was surprising, since this mouse was reported to be null for the Ca_v_1.4 channel protein [22]. Therefore, we next tested to ensure that the ERGs in our mice were identical to those previously published.

**Figure 4:**
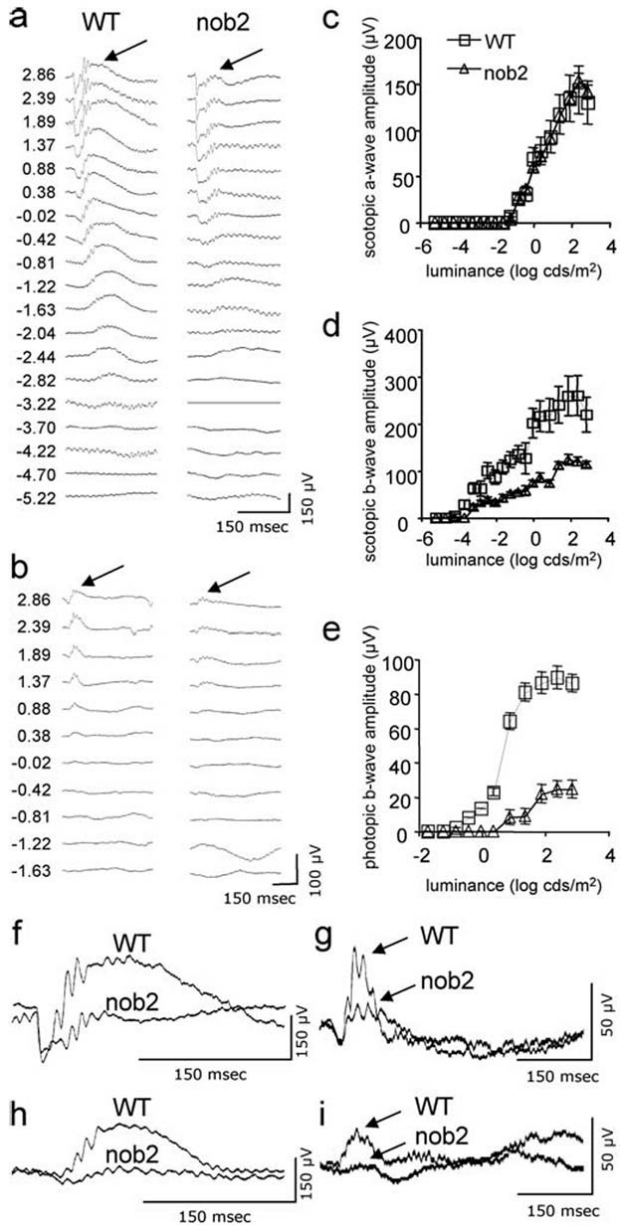
ERG results showing selective inner retina defect in *Cacna1f^nob2^* mice. Representative ERG traces of responses obtained at various intensities under scotopic (panel A) and photopic (panel B) adaptation are given for a *Cacna1f^wt^* (left column) and an age-matched (52 days) *Cacna1f^nob2^* mouse (right column); numbers on left of the traces correspond to the luminances (log cd/m^2^) of the flashes that elicit these responses; arrows point to the b-wave apex. Corresponding amplitudes of the a-wave and b-wave under scotopic conditions are shown in panels C and D, respectively; and of b-wave amplitudes under photopic conditions are shown in panel E. Finally, examples of comparisons of responses obtained from *Cacna1f^wt^* and *Cacna1f^nob2^* are illustrated by superimposing the respective traces obtained from these two mouse types. Responses to high-intensity stimuli (1.89 cd/m^2^) are shown in panels F (scotopic adaptation) and G (photopic adaptation), and responses to low-intensity stimuli are shown in panels H (−0.81 cd/m^2^ under scotopic adaptation) and I (0.38 cd/m^2^ under photopic adaptation).

ERG recordings showed a selective b-wave defect in *Cacna1f^nob2^*, compared with that in age-matched *Cacna1f^wt^* mice (Figure 4), as previously reported [22], [29]. Under both scotopic and photopic adaptation conditions, the amplitude of the b-wave was selectively diminished. The intensity-response curves for the scotopic a-wave, as well as maximal a-wave amplitudes and thresholds (minimum luminance to reach criterion amplitude of 20 µV), were comparable in our mutant and wild type mice. In contrast, however, the b-waves in mutant and wild type mice were very different. The proportion of maximal b-wave remaining in *Cacna1f^nob2^* compared with age-matched *Cacna1f^wt^* was statistically significantly greater (p<0.05, U-test) under scotopic (0.51±0.11) than under photopic (0.29±0.19) conditions. A typical way to document selective b-wave loss, clinically, is to compare the b/a ratios with values from control subjects: lower ratios mean selective b-wave defects. There was a statistically significant decrease in b/a ratios in mutant compared to wild type mice. The reductions in b/a ratios were more pronounced under photopic (0.99±0.19 in *Cacna1f^nob2^* compared with 3.58±0.81 in *Cacna1f^wt^*; p<0.05, U-test) than scotopic conditions (0.88±0.05 in *Cacna1f^nob2^* compared to 1.94±0.09 in *Cacna1f^wt^*; p<0.05, U-test). In addition, the threshold was higher in *Cacna1f^nob2^* mice under scotopic as well as photopic conditions, and the photopic thresholds in *Cacna1f^nob2^* mice were significantly higher than the scotopic thresholds. One b-wave property that was not affected in the *Cacna1f^nob2^* mice compared with *Cacna1f^wt^* mice was the intensity at which maximal b-wave amplitudes were attained. Finally, while we did not systematically quantify the amplitudes of ERG oscillatory potentials, qualitatively they were depressed in *Cacna1f^nob2^* as compared with those in *Cacna1f^wt^* mice.

Upon histological examination, we found that the structure of retinas from *Cacna1f^nob2^* was disorganized compared to that of *Cacna1f^wt^* mice, with substantially reduced outer plexiform layer thickness and dendritic sprouting of second-order neurons into the outer nuclear layer (data not shown). These findings are consistent with those reported previously in the original *Cacna1f^nob2^* mouse strain [22], [30].

### Cacna1f^nob2^ and Cacna1f^wt^ optokinetic responses (OKR) are similar

Given that our *Cacna1f^nob2^*mice produced mRNA capable of encoding full-length Ca_v_1.4 calcium channels (albeit at reduced levels, with only ∼10% of the mRNA encoding full-length Ca_v_1.4 protein) but still had defects in ERG and retinal structure, it was important to test the consequences of these deficits for vision. Therefore we assessed the ability of the mouse to track moving sine-wave gratings.

**Figure 5:**
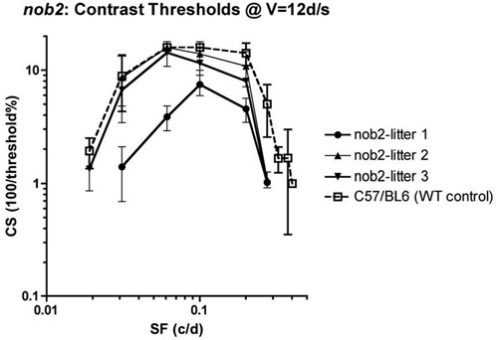
Optokinetic spatial contrast-sensitivity functions for three different *Cacna1f^nob2^* litters and one *Cacna1f^wt^* litter. Mean contrast sensitivities±standard deviation, at drift velocity (V) = 12 degrees per second (d/s) and various spatial frequencies (SF) in cycles per degree (c/d). See Table 2 for further details and statistics.

All *Cacna1f^nob2^* mice tested showed robust optokinetic (more correctly, head-turning or optocollic) responses to moving sine-wave gratings, over a wide range of drift velocities and spatial frequencies (Table 2; Figure 5). Their contrast sensitivities (CS) were maximal at V = 12 degrees/second (d/s), at all spatial frequencies (data for other velocities not shown). The CS functions at V = 12 d/s were identical in litters 2 and 3; CS was maximal at 0.061 cycles/degree (c/d), and declined precipitously at spatial frequencies above 0.2 c/d but more gradually at spatial frequencies below 0.061 c/d. In litter 1, however, CS was maximal at 0.1 c/d and was substantially lower than that of litters 2 and 3 at most spatial frequencies tested (Figure 5). The lower limit of spatial frequencies that the animals could follow was 0.019–0.031 c/d, and the upper limit (“acuity”) was 0.275 c/d, in all three litters. There was no significant difference between the contrast sensitivities of litters 2 and 3 (P>0.05; Student-Neuman-Keuls Multiple Comparisons Test), whereas the contrast sensitivity of litters 2 and 3 (at V = 12 d/s) differed significantly from that of litter 1 (both P<0.001) at most spatial frequencies.

**Table 2:** Optokinetic Response Data for *Cacna1f^nob2^* and *Cacna1f^wt^* mice.

Group (n)	Optimum spatial freq. @ V = 12 d/s	Threshold Contrast (%) at Optimum	Contrast Sensitivity at Optimum	Mean Difference ( 0.061 c/d; V = 12 d/s)	P (2-tailed t-test)
*nob2* - Litter#1 (6)	0.1 c/d	13.39%	7.47	N/A	——
	0.061 c/d	25.9%	3.86	−12.038 vs Litter #3;	<0.001 (highly significant)
				10.458 vs Litter #2	<0.001 (highly significant)
*nob2* – Litter#2 (4)	0.061 c/d,	6.3%	15.9	−1.58, vs Litter #3	>0.05*
*nob2* – Litter #3 (5)	0.061 c/d,	7.0%	14.32	See above	——
*nob2* -Combined Litters#(2+3) (9)	0.061 c/d,	6.7%	15.08 (averaged)	N/A	——
WT (C57/BL6) (6)	0.061 c/d,	6.3%	15.9	0.88 vs Litters(2+3)	0.3454

The contrast sensitivities of *Cacna1f^wt^* mice were maximal at V = 12 d/s, at all spatial frequencies tested (data for other velocities not shown). The CS function at V = 12 d/s was similar to that of *Cacna1f^nob2^* litters 2 and 3 at 0.031–0.1 c/d, but contrast sensitivity remained high at 0.2 c/d and declined precipitously only at spatial frequencies ≥0.275 c/d (Figure 5). The upper limit of spatial frequencies that could be followed by *Cacna1f^wt^* mice was 0.4 c/d, or ∼1.5× the acuity of *Cacna1f^nob2^* mice. The diminished contrast sensitivity seen in the one litter (#1) of *Cacna1f^nob2^* mice has not been seen in any WT mice, regardless of litter or age (S.P. Bonfield, unpublished results).

### Biophysical properties of Ca_v_1.4^wt^ and Ca_v_1.4^nob2^ channels are statistically indistinguishable

**Figure 6:**
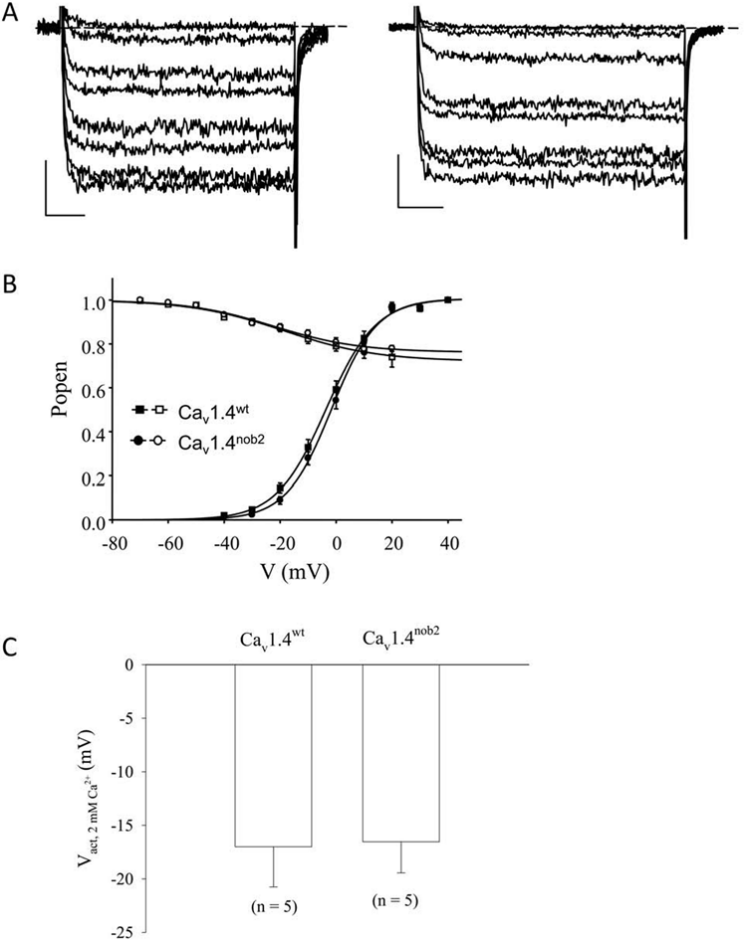
Biophysical properties of Ca_v_1.4^wt^ and Ca_v_1.4^nob2^ channels, coexpressed with β_2a_ and α_2_–δ_1_ subunits in tSA-201 cells. A. Representative current waveforms for Ca_v_1.4^wt^ (left) and Ca_v_1.4^nob2^ (right) recorded with 20 mM Ba^2+^ external saline. Horizontal scale bars denote 25 ms, and vertical scale bars 25 pA. B. Average activation (filled symbols) and inactivation (hollow) symbols for Ca_v_1.4^wt^ (squares) and Ca_v_1.4^nob2^ (circles) recorded with 20 mM Ba^2+^ external saline. Average activation parameters from 11 Ca_v_1.4^wt^ cells and 13 Ca_v_1.4^nob2^ cells are: V_act, wt_ = −3±4 mV, V_act, nob2_ = −1±4 mV (n = 13); G_max, wt_ = 4±3 nS and G_max, nob2_ = 3±1 nS; S_wt_ = 9±1 mV and S_nob2_ = 8.2±0.8 mV. These values are stastically identical, and are summarized in Table 3. Average inactivation parameters from these cells are V_inact, wt_ = −18±11 mV and V_inact, nob2_ = −22±10 mV, with a large fraction of non-inactivating current for both channels. These values statistically identical and are summarized in Table 3. C. Average half-inactivation potentials for channels recorded with 2 mM Ca^2+^ as charge carrier. Currents were substantially smaller than with 20 mM Ba^2+^, but were distinguishable from background noise, and were obtained using a ramp protocol identical to that previously reported [31], obtained by ramping voltage from −100 mV to +100 mV over 500 ms. Values were V_act, wt_ = −17±8 mV (average peak current size −9±4 pA) and V_act, nob2_ = −17±6 mV (average peak current size −9±3 pA). The shift observed with switching from 20 mM Ba^2+^ to 2 mM Ca^2+^ as external charge carrier is similar to that we have previously reported for the human Ca_v_1.4 channels [16].

Next, we tested whether the Cav1.4^nob2^ channel could support ionic currents. Standard whole-cell electrophysiological recordings were obtained from tsA-201 cells transfected with mouse Ca_v_1.4*^wt^* or Ca_v_1.4*^nob2^* (along with rat β_2a_ and α_2_–δ_1_) constructs. With 20 mM Ba^2+^ as charge carrier, the average activation and inactivation parameters for Ca_v_1.4^nob2^ were not significantly different from those of Ca_v_1.4^wt^ (p>0.05, t-test; Table 3; Figure 6). Also, with 2 mM Ca^2+^ as the charge carrier and using ramp protocols [31], [32], no differences were observed in the activation properties between the two clones (see inset, Fig 6).

**Table 3:** Summary of biophysical properties of mouse Ca_v_1.4^wt^and Ca_v_1.4^nob2^ constructs recorded with 20 mM Ba^2+^ as charge carrier.

	V_act_ (mV)	G_max_ (nS)	S (mV)	E_rev_ (mV)	V_inact_ (mV)	z
Ca_v_1.4^wt^	−3±4 (11)	4±3 (11)	9±1 (11)	44±6 (11)	−18±11 (11)	1.9±0.6 (11)
Ca_v_1.4^nob2^	−1±4 (13)	3±1 (13)	8.2±0.8 (13)	45±6 (13)	−22±10 (12)	3±3 (12)
Ca_v_1.4^wt^ (s.s.)	−1±6 (4)	4±3 (4)	9±1 (4)	44±7 (4)	−22±9 (4)	2.0±0.6 (4)
Ca_v_1.4^wt^ (s.s.+PD98059)	1±2 (4)	3.4±0.8 (4)	8.4±0.8 (4)	46±4 (4)	−17±9 (4)	1.8±0.4 (4)
Ca_v_1.4^nob2^ (s.s)	−4±3 (4)	2.1±0.3 (4)	8.7±0.3 (4)	41±6 (4)	−28±6 (4)	2.4±0.7 (4)
Ca_v_1.4^nob2^ (s.s.+PD98059)	−5±2 (5)	2.0±0.9 (5)	8.4±0.3 (5)	41±6 (5)	−18±5 (5)	1.7±0.5 (4)

s.s. denotes serum starvation for 2 hours; no statistical difference is observed between mouse Ca_v_1.4^wt^ and Ca_v_1.4^nob2^, or between the constructs when currents were recorded in the absence or presence of 20 µM PD98059 following two hours of serum starvation (p>0.05, one-way ANOVA).

The Ca_v_1.4^wt^ N-terminus contains a putative site for phosphorylation by mitogen-activated protein (MAP) kinase that is not present in Ca_v_1.4^nob2^ (serine residue in PEPSPAN region of wild type clone (Figure 2C) identified by ELM – Functional Sites in Proteins, http://www.elm.eu.org). To test whether this predicted phosphorylation site is differentially phosphorylated, thereby causing a functional difference between the two clones, we blocked MAP kinase activity in serum-starved cells by treating them for 2 hours with 20 µM PD98059 (a specific inhibitor for MAP kinase kinase; e.g., [33], [34]). PD98059 did not significantly alter the biophysical properties of either Ca_v_1.4^wt^ or Ca_v_1.4^nob2^ channels (p>0.05) in comparison with those that were recorded in the absence of the inhibitor (Table 3).

### N-terminus from Ca_v_1.4^wt^ and not Ca_v_1.4^nob2^ can interact with filamin proteins

**Figure 7:**
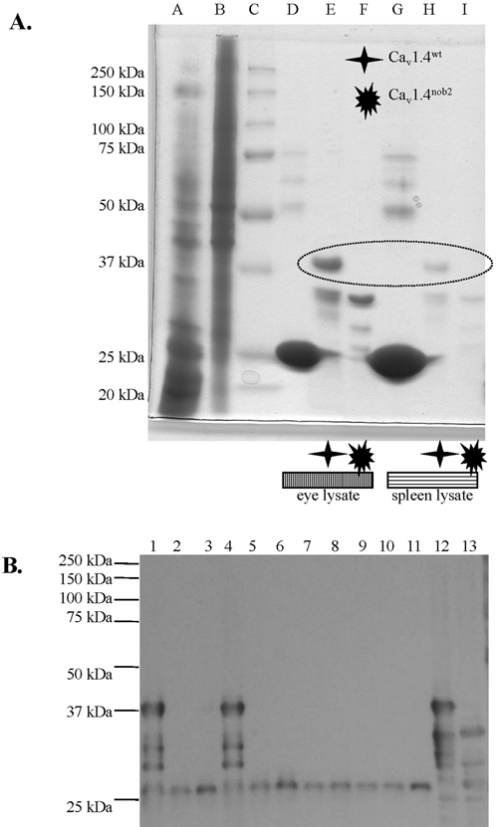
A. Representative Coomassie Brilliant Blue-stained 10% PAGE gel from three separate experiments. N-termini from Ca_v_1.4^wt^ or Ca_v_1.4^nob2^ were fused to glutathione S-transferase and purified on glutathione beads. Lysates from eye (lanes D–F) or spleen (G–I) were then incubated with the beads, and subsequently washed. Unpurified eye or spleen lysates (5 µL) or purified beads+lysates (40 µL) were loaded onto the gel. Lanes are as follows: A: unpurified eye, B: unpurified spleen, C: protein ladder, D: glutathione-sepharose beads+eye lysates, E: glutathione-sepharose beads+eye lysates+GST-Ca_v_1.4 N-terminus, F: glutathione-sepharose beads+eye lysates+GST-Ca_v_1.4^nob2^ N-terminus, G: glutathione-sepharose beads+spleen lysates, H: glutathione-sepharose beads+spleen lysates+GST-Ca_v_1.4 N-terminus, I: glutathione-sepharose beads+spleen lysates+GST-Ca_v_1.4^nob2^ N-terminus. A prominent band of M_r_ slightly larger than 37 kDa is observed in lanes E and H, but is absent from lanes F and I. This band was interpreted as a filamin A protein fragment. B. GST-fused N-termini from Ca_v_1.4^wt^ or Ca_v_1.4^nob2^ were incubated with HA-tagged C-termini of filamin A or filamin B. Only GST-Cav1.4^wt^ N-terminus was capable of interacting with either filamin A or filamin B. Lanes 1-3 correspond to filamin A (with GST-Cav1.4^wt^, GST-Cav1.4^nob2^, and GST, respectively); lanes 4–6 correspond to filamin B (with GST-Cav1.4^wt^, GST-Cav1.4^nob2^, and GST, respectively); lanes 7 and 8 to filamin A and B, respectively (no GST construct); lanes 9–11 GST-Cav1.4^wt^, GST-Cav1.4^nob2^, and GST, respectively (with no filamin constructs); lane 12 is unpurified GST-Cav1.4^wt^ lysate; lane 13 is unpurified GST-Cav1.4^nob2^ lysate.

To test whether Ca_v_1.4^wt^ and Ca_v_1.4^nob2^ constructs differentially interact with cellular proteins, we utilized a biochemical pull-down assay. As shown in Figure 7A, a band of approximately 37 kDa was pulled down by Ca_v_1.4^wt^ but not Ca_v_1.4^nob2^ N-termini. This band was excised, and the protein was subsequently identified as filamin A by means of LC/MS/MS.

To investigate these interactions further, both -N-termini and filamin proteins were used as biochemical pull-down ligands. Filamin A and filamin B constructs with an HA tag were mixed with the GST- Ca_v_1.4^wt^ and GST-Ca_v_1.4^nob2^ lysates. As shown in Figure 7B, the N-terminus of the Ca_v_1.4^wt^ channel protein interacted with the C-termini of both filamin A and filamin B, whereas the N-terminus of the Ca_v_1.4^nob2^ channel protein did not.

## Discussion

Taken together, our results suggest that alternative splicing within the ETn element inserted into exon 2 of the CACNA1F gene of the *Cacna1f^nob2^* mouse allows for full-length Ca_v_1.4 protein to be produced, and therefore this mouse model is not null for the Ca_v_1.4 calcium channel. These results contrast with those previously published [22].

Because of their highly repetitive sequence, transposable elements have previously been shown to undergo alternative splicing; this allows for full-length protein to be produced, albeit at reduced levels relative to those in WT controls [26], [27]. In agreement with these previous findings (depicted in Figure 1B), we detected differentially spliced mRNA isoforms in the *Cacna1f^nob2^* mouse, with approximately 90% of the mRNA expected to be transcribed to Ca_v_1.4 protein having a premature stop codon in exon 2, and 10% of the mRNA expected to be transcribed to full-length channel protein with a mutated N-terminus (Figure 2C). Full-length protein was detected in spleen samples with Western blots (Figure 3), confirming that the alternatively spliced mRNA species is capable of producing Ca_v_1.4 channel proteins with a molecular mass of ∼230 kDa. We attempted to detect Ca_v_1.4 channel protein in sections of mouse retina; however our antibody is directed against the C-terminus of the human protein, which shares only ∼50% sequence homology with the mouse protein (rendering the antibody only weakly cross-reactive and therefore unsuitable for immunohistochemistry in mouse). Therefore, it was necessary to use concentrated protein lysates from spleen on Western blots, where Ca_v_1.4 protein appears to be greatly enriched [16]. While it is possible no functional Ca_v_1.4 protein is present in the *Cacna1f^nob2^* mouse retina, the reduced protein levels in the retina may have accounted for the apparent background labeling in previous reports (for example, the faint labeling in Figure 1b from Chang et al., 2006). Thus, the CSNB2 phenotype in the *Cacna1f^nob2^* mouse could arise simply from reduced Ca_v_1.4 protein levels, rather than complete knockout of protein. This hypothesis is supported by previous studies, which have shown that R508Q and L1364H mutations of *CACNA1F* linked to CSNB2 reduce the amount of functional protein in the membrane, without significantly altering the biophysical properties of the channel [15].

Since our findings differ substantially from those reported previously [22], it was necessary to ensure that the *CACNA1F* gene mutation in the *Cacna1f^nob2^* mouse line that we were testing was the same as that originally described. Therefore, we sequenced genomic DNA, obtained both from our in-house colony and as supplied by the Jackson Laboratory. In both cases the genomic sequence confirmed the presence of the ETn element inserted into exon 2 of the *CACNA1F* gene. Additionally, the mice we tested showed alterations in the ERGs (Figure 4) that were consistent with previous findings [22], [30]. Taken together, these results suggest that the *CACNA1F* gene mutation in the mice we were testing was identical to that in the line originally described, and that the inherited defect in our colony was not the result of a random genetic mutation or a deletion of the ETn element from exon 2.

As shown in Figure 4, the *Cacna1f^nob2^* mouse retina is characterized by a functional defect that selectively affects the light-driven activation of neurons in the inner retinal layers; this is supported by the depression of the ERG b-wave (reflecting diminished modulation of activity in depolarizing bipolar cells), while the a-wave amplitudes are unaffected (indicating that photoreceptor activity is relatively unaffected). The partial preservation of the b-wave in these mice is reminiscent of the incomplete form of CSNB (CSNB2 due to *CACNA1F* mutations), in contrast to the complete absence of a recordable b-wave in the complete form of CSNB (CSNB1; [35], [36]. As previously reported in *Cacna1f^nob2^* mice, *CACNA1F* mutations in humans cause ERG b-wave reductions under both dark-adapted (scotopic) and light-adapted (photopic) conditions. Another similarity between *Cacna1f^nob2^* mice and CSNB2 patients is the depression of the OPs under scotopic conditions. Finally, both humans with *CACNA1F* mutations and *Cacna1f^nob2^* mice have normal photopic and scotopic a-wave amplitudes [35], [36], (Figure 5). There is evidence that decreases in b-wave amplitude might lead to increases in a-wave amplitudes. For instance, pharmacological blockade of mGluR6 receptors (with intravitreal injections of L-glutamate or APB) does completely abolish the b-wave (also known as PII) and reveal photoreceptor activity (also known as PIII) over its full time course, and does increase the amplitude of the a-wave/PIII. However, in cases of complete b-wave loss due to nyctalopin mutations, a-wave amplitudes are normal under both scotopic and photopic adaptation. So the outcome, whether b-wave reductions affect a-wave amplitudes, might depend on the mechanism by which the b-wave is abolished or decreased in amplitude. The possibility that the mutated Ca_v_1.4 channel might lead to reduced a-wave amplitudes, and that these reductions might be compensated by the indirect effect of increased a-wave amplitudes due to reduced b-wave amplitudes, does exist, but we have no experimental evidence to verify whether this is the case.

Previously, *Cacna1f^nob2^* mice were found to have a reduced b-wave, and morphological studies of the *Cacna1f^nob2^* retina showed a reduced OPL thickness and sprouting of second-order neurons into the ONL. Therefore, we expected that the visual performance of *Cacna1f^nob2^* mice would be greatly reduced or absent, as in *Cacna1f^G305X^* mice [37]. Quite unexpectedly, our *Cacna1f^nob2^* mice responded robustly to a full range of sine-wave gratings, showing only slight differences from the optokinetic performance of WT-C57/BL6 mice. The major difference in visual performance of the best-performing *Cacna1f^nob2^* mice was that their CS functions cut off more sharply than those of *Cacna1f^wt^* mice at spatial frequencies ≥0.2 c/d, resulting in an optokinetic acuity of 0.275 c/d (Figure 6; Table 2). As a result, the spatial acuity of *Cacna1f^nob2^* mice is about two-thirds of the acuity of C57BL6 mice, which is 0.4 c/d [37].

Thus, our behavioral results show that the *Cacna1f^nob2^* mouse has good vision (at least for the optokinetic response), despite its severe ERG phenotype and morphologically abnormal outer retina. The relatively good vision of *Cacna1f^nob2^* mice contrasts starkly with the lack of optokinetic responses in a targeted *CACNA1F* knockout, the G305X mutant [37], suggesting that the *Cacna1f^nob2^* phenotype is not due solely to a truncation or loss-of-function mutation. Since our *Cacna1f^nob2^* animals perform nearly as well as wild type controls, it is clear that in spite of the histological and electroretinographic evidence of severe retinal dysfunction, the *Cacna1f^nob2^* mouse maintains at least some nearly normal visual processing in proximal levels of the retina. Our finding that about 10% of whole-retinal *Cacna1f^nob2^* mRNA is expected to encode a full-length channel protein similar to the wild type (whereas 90% carries the in-frame stop codon) raises the question whether every photoreceptor cell makes 10% WT-like protein, or 10% of photoreceptor cells make 100% wild type-like protein (or some combination of these). On the one hand, the loss of synapses in the OPL and the sprouting of dendrites into the ONL point to significant defects in the synthesis of Ca_v_1.4 protein, and consequently of transmitter release, in the vast majority of photoreceptor cells; this suggests that nearly all *Cacna1f^nob2^* photoreceptor cells are making insufficient amounts of functional channel protein. On the other hand, the retention of normal contrast sensitivity and near-normal optokinetic acuity under photopic conditions indicates that cone pathways are only minimally affected. We suggest, therefore, that the wild type-like transcript is expressed mainly or exclusively in cone photoreceptors, accounting for the relative sparing of cone transmission as seen in the optokinetic response, and that the mutant transcript is expressed mainly or exclusively in rod photoreceptors, accounting for the striking loss of synaptic terminals and consequent thinning in the OPL. The discrepancy, between retention of cone function in the optokinetic response and loss of cone signals in the ERG, may be due to differential processing of cone information or mutation-dependent changes in the retinal circuits responsible for the OKR and ERG. The experiments that would be required to test this hypothesis are beyond the scope of the present study.

While full-length Ca_v_1.4 protein is produced (albeit at presumably reduced levels) in the *Cacna1f^nob2^* mouse, the encoded amino acid sequence differs from that in the wild type by approximately 20 residues in the N-terminus of the protein (Figure 2C). As shown in Figure 6, the electrophysiological properties of Ca_v_1.4^nob2^ are not significantly different from those of Ca_v_1.4^wt^ when expressed in tsA-201 cells, with either 20 mM Ba^2+^ or 2 mM Ca^2+^ as charge carrier, suggesting that the differences between *Cacna1f^nob2^* and *Cacna1f^wt^* mice do not arise from changes in the activation or inactivation characteristics of the Ca_v_1.4 channels. Interestingly, within the span of amino acids that differ between the mutant and wild type proteins, the Ca_v_1.4^nob2^ channel sequence lacks a putative MAP kinase phosphorylation site that is present in the wild type protein. PD98059, a specific inhibitor of MAPKK, did not have a differential effect on the biophysical properties of either Ca_v_1.4^nob2^ or Ca_v_1.4^wt^, suggesting that the difference in biophysical properties of these channels is not due to a difference in phosphorylation within these 20 amino acids.

However, as shown in Figure 7, the mutant and wild type N-terminal regions do differ in that they interact with different proteins, since the N-terminal GST-fusion protein for the Ca_v_1.4^wt^ sequence pulls down filamin protein, whereas the Ca_v_1.4^nob2^ peptide does not. Filamins are a family of cytoskeleton proteins (200–300 kDa) that regulate and crosslink filamentous actin. They are highly sensitive to proteolysis, and numerous fragments of varying size, from 10 kDa to 280 kDa, have been identified [38]; reviewed in [39]. Calpain 3 has been shown to cleave full-length filamin A (∼260 kDa), yielding one identified product of ∼220 kDa [40]; the other cleavage product, which is expected to have a molecular mass of ∼40 kDa, thus may account for the filamin fragment identified in Figure 7A. Additionally, several previous studies have demonstrated that interactions of ion channels with filamin proteins result in enhanced targeting of the ion channels to the plasma membrane accompanied by increased current densities, specifically for Kir2.1 (binds to C-terminus of FLNA; [41]), Kv4.2 and Kv4.3 [42], and CFTR [43]–[46]. In the case of Kv4.2, a proline-rich region of the channel was identified as necessary for the interaction, with only four amino acids (PTPP) required for binding [42]. In our case, the wild type sequence of the 20 amino-acid region unique to the N-terminus of Ca_v_1.4^wt^ contains six proline residues, while that of Ca_v_1.4^nob2^ only contains one; furthermore, at the boundary of this region with the downstream N-terminal sequence, the wild type clone has a PGPP motif (recall PTPP motif for Kv4.2), while the Ca_v_1.4^nob2^ construct has an NRPP motif. While we did not map the exact residues in this 20 amino acid region necessary for this interaction, these data suggest that the CSNB2-like phenotype of the *Cacna1f^nob2^* mouse may result not only from lower Ca_v_1.4-mediated calcium-current densities, due to the diminished production of full-length protein, but also to a failure of targeting of the mutant protein to the plasma membrane, due to its inability to interact with filamin. Since filamin proteins are present in most cell types, any effects on current densities may be masked in our overexpression (transfected cell) system, but such effects could be profound in the highly regulated environment of photoreceptor cells.

## Methods

*Cacna1f^nob2^* (AXB6/pgnJ, stock #001678) mice were purchased from Jackson Laboratory (Bar Harbor, ME, USA). A colony was established by breeding *Cacna1f^nob2^* mice with in-house C57BL/6 mice at the University of Calgary Health Sciences Animal Resource Centre. One male *Cacna1f^G305X^* mouse was obtained from Dr. N.T. Bech-Hansen (University of Calgary). PCR was used to confirm the genotype of all the mice used in this study. All experimental protocols were approved by University of Calgary Animal Care Committee, in accordance with guidelines established by the Canadian Council of Animal Care and the ARVO Statement for the Use of Animals in Ophthalmic and Visual Research.

### RT-PCR, splice variant generation, and genomic analysis

Total RNA was isolated from a whole mouse eye (minus lens), using Trizol (Invitrogen). To synthesize cDNA, 5 µL of isolated RNA was added to oligo-dT primers along with Superscript II reverse transcriptase (Invitrogen) and incubated according to the manufacturer's instructions. To probe for *CACNA1F* transcripts, corresponding primers were synthesized flanking the ETn element in exon 1 (RR44) and exon 2 (RR45) or exon 3 (RR46) (Table 1; Figure 1A). For amplification, 2 µL of cDNA and Hot Start Taq (Qiagen) were used according to the manufacturer's instructions, and the reaction product was analyzed on a 1.5% agarose gel; DNA bands were isolated using a gel extraction kit (Qiagen), cloned into pGEM-T-easy (Stratagene), and then sequenced to confirm identity.

Full length mouse Ca_v_1.4 cDNA (Ca_v_1.4^wt^; [47] was subcloned into pCDNA3.1zeo, and the Ca_v_1.4^nob2^ splice variant was synthesized using site-directed mutagenesis and Not I and Spe I restriction enzymes. After the sequences had been confirmed, constructs were transfected into an expression system for electrophysiological analysis (see below).

### Biochemistry

For analysis of Ca_v_1.4^wt^ or Ca_v_1.4^nob2^ expression by polyacrylamide gel electrophoresis, protein concentrations in spleen lysates were determined using a DC protein assay kit (BioRad), and equal total protein was loaded into each lane. Ca_v_1.4 proteins were detected using 0.7 µg of an affinity purified rabbit polyclonal anti-Ca_v_1.4 antibody (1∶1000 dilution; [16]). The membranes were then washed, incubated with secondary HRP-conjugated anti-rat IgG antibody (1∶5000 dilution, GE Healthcare) and detected using standard ECL methods. Spleen lysate from a *Cacna1f^G305X^* mouse, which does not make complete Ca_v_1.4 channel protein [21], was prepared similarly as a negative control. For the Western blots, 4 separate experiments (n = 4) were performed and revealed the same results.

PCR was used to amplify the N-termini of mouse Ca_v_1.4^wt^ and Ca_v_1.4^nob2^ from their appropriate cDNA templates, using primers CDRR69 (with incorporated BamHI restriction site; Table 1) and CDRR70 (with incorporated EcoRI site, Table 1). The fragment was ligated into the T-easy vector system (Promega). After the PCR product sequence had been verified, the N-terminal fragments were excised (BamHI, EcoRI) and subcloned into the pGEX-5X-1 GST Fusion System (Pharmacia). The resulting GST-fusion proteins were purified using glutathione sepharose beads (Amersham) according to the manufacturer's instructions. A 50 µL aliquot of beads was mixed with 20 µL eye or spleen lysate overnight at 4°C (whole eyes minus lens, from 7 C57/BL6 mice, were pooled and homogenized in 200 µL total volume; spleens from these mice were pooled and homogenized in 1 mL total volume), beads washed, and then proteins resolved on a 10% polyacrylamide gel stained with Coomassie Brilliant Blue. These experiments were performed 3 separate times (n = 3) resulting in identical results. Protein bands of interest were excised from the gel, and the protein sequence was determined using LC/MS/MS (Southern Alberta Mass Spectrometry Centre).

Mouse filamin cDNA was obtained from OpenBiosystems. PCR was used to amplify (past repeat 23) and add an HA tag onto the C-terminal regions of filamins A and B. Each fragment was ligated into the T-easy vector system (Promega), the sequence was confirmed, and the fragment was subcloned into the expression vector pCDNA3.1neo. The resulting construct was transfected into tsA-201 cells (transfection details below). After a two-day incubation the cells were lysed on ice with lysis buffer (300 mM NaCl, 50 mM Tris pH 7.5, 0.1% Triton X-100) containing a protease-inhibitor cocktail (complete Mini EDTA-free, Roche). The lysate was incubated with sepharose G beads (Amersham) and Anti-HA High Affinity antibody (Roche) at 4°C for 4 hours. The beads were then washed and incubated with 1 ml of GST- Ca_v_1.4^wt^ or GST- Ca_v_1.4^nob2^ cell lysate, overnight at 4°C. The beads were washed again, and then proteins were resolved using PAGE (12% gel), transferred to a PVDF membrane, and probed with Anti-GST antibody (1∶2000 dilution, GE Healthcare). Finally the membranes were washed, incubated with HRP-conjugated anti-rat-IgG antibody (1∶5000 dilution, GE Healthcare) and detected using standard ECL methods. These experiments were performed 3 independent times (n = 3) and had the identical results.

### Tissue culture and transfection

Culturing and transfection of tsA-201 cells via the calcium phosphate method have been previously described by us in detail [16]. Briefly, tsA-201 cells were maintained in DMEM supplemented with 10% FBS and 50 U/mL penicillin-streptomycin. Cells were grown to 80% confluency (37°C, humidified, 5% CO_2_), dissociated enzymatically (trypsin-EDTA), and then plated at 10% confluency on glass cover slips. After recovering for 8 hours, cells were transfected using standard calcium phosphate techniques (in all cases 6 µg each of cDNA encoding mouse α_1_, rat β_2a_, and rat α_2_–δ_1_ subunits, and 1 µg pIRES transfection marker). Twelve hours later, cells were washed with fresh media and then moved to 29°C (5% CO_2_, humidified) for two to three days before evaluation by the whole-cell patch clamp technique.

### Electrophysiology

Electrophysiological data were acquired using an Axopatch 200B amplifier (Axon Instruments, Union City, CA) linked to a personal computer with Digidata 1322A interface, using pClamp 9.1 (Axon) software. In all cases, pipette capacitance and series resistance were compensated (series resistance by 80%). Currents were filtered at 1 kHz and digitized at 2 kHz. Prior to recording, individual cover slips were transferred to a 3 cm culture dish filled with bath recording solution (in mM: 20 BaCl_2_ (or 2 CaCl_2_ for ramp protocols), 65 CsCl, 40 TEA-Cl, 1 MgCl_2_, 10 glucose, 10 HEPES, pH 7.20 with TEA-OH). Pipettes (BF-150-86 borosilicate glass, Sutter Instruments, Novato CA) were pulled on a P-87 microelectrode puller (Sutter Instruments) and fire-polished with an MF-830 microforge (Narishige, Japan) to a resistance of 1–4 MΩ when filled with recording solution (in mM: 108 CsCH_3_SO_3_, 4 MgCl_2_, 9 EGTA, 9 HEPES pH 7.20 with CsOH).

Current-voltage relations were obtained by holding cells at −100 mV before stepping to various test potentials for 150 ms, typically at a frequency of 0.2 Hz. Ramp experiments were obtained by changing membrane voltage from −100 mV to +100 mV over 500 ms. Whole-cell current-voltage relations were fitted with Equation 1, where *I* denotes peak current amplitude, *V* is the test potential, *V*_act_ is the half-activation potential, *S* is a slope factor, *G*_max_ is maximum chord/slope (whole-cell) conductance, and *E*_rev_ is reversal potential.
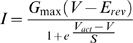
(1)


Inactivation-voltage relations were obtained by holding cells at −100 mV, depolarizing to a test potential of +20 mV for 50 ms, stepping back to −100 mV for 1 ms before initiating a 10 s conditioning pulse to various potentials, stepping back to −100 mV for 1 ms, and initiating a second test pulse to +20 mV for 50 ms. The degree of inactivation was determined as the ratio of the second test pulse to the first test pulse. Inactivation-voltage relations were fitted with Equation 2, where *I* is the degree of inactivation, *V* is the conditioning potential, *V*_inact_ is the half-inactivation potential, *z* is a slope factor reflecting effective gating charge, and *x* represents the non-inactivating fraction of current. Since experiments were typically carried out at room temperature, R (universal gas constant), F (Faraday constant), and T (absolute temperature) simplify to a value of 25.6 mV in this equation (ie. RT/F = 25.6 mV).
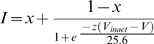
(2)Electrophysiological data were analyzed using Clampfit9.1 software (Axon Instruments, Union City, CA) and SigmaPlot2000 (Jandel Scientific, Chicago, IL).

PD98059, a mitogen-activated protein (MAP) kinase inhibitor, (Tocris Biosciences) was dissolved in DMSO at a stock concentration of 25 mM, and then diluted into the final recording solution immediately before recording. Control and test cells were serum-starved for 2 hours in the presence or absence of inhibitor (see for example, [34], [48]–[50]) prior to electrophysiological analysis. Control (with DMSO vehicle) and drug (20 µM) saline solutions were delivered by a gravity-driven microperfusion system.

### ERG recordings

ERGs were recorded from *Cacna1f^nob2^* (n = 6) and age-matched *Cacna1f^wt^* (n = 5) mice, as previously described [51]. Briefly, after overnight dark-adaptation, mice were prepared for bilateral recordings under dim red light. While under anesthesia (xylazine 10 mg/kg i.p; ketamine 150 mg/kg i.p.) the mouse body temperature was monitored with a rectal probe and maintained at 38°C using a homeothermic blanket. Both pupils were dilated using 1% tropicamide. A drop of methylcellulose, applied on each cornea, prevented dehydration and allowed electrical contact with the recording electrode (gold wire loop). A pair of 25-gauge platinum needles inserted subdermally behind each eye served as reference electrodes. Amplification (1–1000 Hz bandpass), stimulus presentation, and data acquisition were provided by the Espion E 2 ERG system (Diagnosys LLC, Lowell, MA). First, scotopic intensity-response functions were determined using single flashes (6500K, 10 µs duration) presented to dark-adapted animals at nineteen increasing intensity steps from −5.22 to 2.86 log cds/m^2^. The inter-stimulus-interval (ISI) was increased progressively from 5 sec (at the lowest stimulus intensity) to 2 minutes (at the highest stimulus intensity), so as to minimize rod photopigment bleaching and desensitization. The amplitude of the b-wave was measured from the a-wave negative peak to the b-wave positive apex, and not to the peak of oscillatory potentials (OPs), which can exceed the b-wave apex [52]. After 10 min photopic adaptation (30 cd/m^2^ background), cone-driven intensity-response functions were obtained, using single flashes (6500K, 10 µs duration) presented at eleven increasing intensity steps from −1.63 to 2.86 log cds/m^2^. The time interval between steps was 10 seconds, and each stimulus was presented 6 times at 5 sec intervals. Responses were averaged for the six flashes at each intensity. For data analysis, responses from both eyes were considered, therefore the values in the graphs represent average±standard deviations for n = 12 *Cacna1f^nob2^* and n = 10 *Cacna1f^wt^* eyes.

#### Optokinetic (Head-Turning or Optocollic) Responses

Contrast sensitivity functions were obtained for optokinetic responses to moving sine-wave gratings, using the virtual optomotor apparatus, OptoMotry™ (Cerebral Mechanics, Lethbridge, AB, Canada; [53], [54]). Briefly, three sets of *Cacna1f^nob2^* littermates of either sex (litter #1: n = 6; litter #2: n = 4; litter #3: n = 5), or their wild type background littermates (n = 6), aged 90–110 days, were placed individually on a 5 cm platform mounted in the middle of a closed testing chamber enclosed by four 17-inch LCD computer monitors (model 1703FP; Dell, Phoenix, AZ). We observed head-following responses by means of a digital video camera mounted above the platform in the lid of the chamber. Horizontally drifting sine-wave gratings, drifting to either the left or right, were presented at various spatial frequencies (0.019 cycles/degree (c/d) to 0.3 c/d) and drift velocities (6, 12, 18, 24, 30 and 36 degrees/second (d/s), for most spatial frequencies). Contrast threshold, defined as the lowest contrast at which the animal could follow the moving grating reliably (Michelson contrast: Equation 3, where L_max_ and L_min_ are the maximum and minimum luminances of the monitors), was determined by a modified staircase procedure and a two-alternative forced-choice paradigm. Contrast sensitivity was defined as the inverse of contrast threshold (100/threshold % contrast), and acuity was defined as the highest spatial frequency at which the animal could follow the drifting grating at 100% contrast.
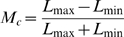
(3)


Contrast, spatial frequency, and rotation velocity are expressed here as the computer settings. True contrast and luminance at the center of the animal's viewing platform were measured with a Minolta LS-110 Luminance Meter, operating as a spot photometer with a 1 degree acceptance angle. The mean luminance was 55 cd/m^2^ for a 1.0 c/d sine-wave grating of maximum contrast moving at 12 d/s, and the mean luminances of “black” and “white” (i.e., the maximum and minimum luminances), determined with a stationary 0.011 c/d grating at 100% contrast, were 3.98 cd/m^2^ and 104.2 cd/m^2^, respectively. Thus, the actual Michelson contrast range was 3.8–100%; contrast thresholds, and the related contrast sensitivities reported in this paper, were not corrected for this small discrepancy from the instrument's contrast settings of 0–100%. Mean luminance of the gratings varied from 27.5 to 110 cd/m^2^ with distance from the monitor screens, as the animal moved freely on the platform.

### Statistics

Statistical analyses were performed using either SigmaStat2.03 (SSI, Richmond, CA), or Prism™ or InStat™ (GraphPad Software Inc., San Diego, CA, USA). Unless otherwise stated, numbers shown are mean±standard deviation, and numbers in parentheses denote the number of experiments ( = number of animals tested). Significant differences are denoted by * (p<0.05). For optokinetic responses, a one-way ANOVA (InStat™) was used to determine whether significant differences existed among the data gathered for the three litters.
